# Evaluation of cosmetic outcomes in breast reconstruction patients undergoing radiotherapy using an anomaly generative adversarial network model

**DOI:** 10.1038/s41598-024-66959-1

**Published:** 2024-07-10

**Authors:** Choong-won Lee, Kyung Hwan Shin, Ji Hyun Chang, Bum-Sup Jang

**Affiliations:** 1 Department of Radiation Oncology, Seoul National University Hospital, Seoul, South Korea; 2 Department of Radiation Oncology, Seoul National University College of Medicine, Seoul, South Korea; 3 Institute of Radiation Medicine, Seoul National University Medical Research Center, Seoul, South Korea

**Keywords:** Breast cancer, Radiotherapy

## Abstract

Considering the rising prevalence of breast reconstruction followed by radiotherapy (RT), evaluating the cosmetic impact of RT is crucial. Currently, there are limited tools for objectively assessing cosmetic outcomes in patients who have undergone reconstruction. Therefore, we validated the cosmetic outcome using a previously developed anomaly Generative Adversarial Network (GAN)-based model and evaluated its utility. Between January 2016 and December 2020, we collected computed tomography (CT) images from 82 breast cancer patients who underwent immediate reconstruction surgery followed by radiotherapy. Among these patients, 38 received immediate implant insertion, while 44 underwent autologous breast reconstruction. Anomaly scores (AS) were estimated using an anomaly GAN model at pre-RT, 1st follow-up, 1-year (Post-1Y) and 2-year (Post-2Y) after RT. Subsequently, the scores were analyzed in a time-series manner, considering reconstruction types (implant versus autologous), RT techniques, and the incidence of major complications. The median age of the patients was 46 years (range 29–62). The AS between Post-1Y and Post-2Y demonstrated a positive relationship (coefficient 0.515, P < 0.001). The AS was significantly associated with objective cosmetic indices, namely Breast Contour Difference (P = 0.009) and Breast Area Difference (P = 0.004), at both Post-1Y and Post-2Y. Subgroup analysis stratified by type of breast reconstruction revealed significantly higher AS values in patients who underwent prosthetic implant insertion compared to those with autologous reconstruction at all follow-up time points (1st follow-up, P = 0.001; Post-1Y, P < 0.001; and Post-2Y, P < 0.001). A threshold AS of ≥ 1.9 was associated with a 10% predicted risk of developing major complications. The feasibility of an AS generated by a GAN model for predicting both cosmetic outcomes and the likelihood of complications following RT has been successfully validated. Further investigation involving a larger patient cohort is warranted.

## Introduction

Despite its initial development in 2007, BCCT.core remains the sole tool for objectively evaluating cosmetic outcomes in breast cancer patients, leading to its continued use. Given that BCCT.core is the only established modality for evaluating aesthetic outcomes in postoperative breast cancer patients, it has been widely adopted in research, including recent prospective trials^1–3^. While developers proposed adopting 3D images for future versions, no clear benefit was outlined^4^. However, its reliability is in question due to discrepancies between patient self-assessments (69.2% to 74.8%) and clinician assessments (25% to 83%).^5^ Additionally, BCCT.core's applicability is limited for patients underwent breast-conserving surgery (BCS). This limitation arises because the software's objective indices are primarily designed for patients who have undergone breast-conserving surgery.

GAN model is a recently emerging novel technique widely adopted in field of imaging due to its exceptionally plausible ability to detect abnormalities between normal images by deep learning. Building upon our prior work establishing the feasibility of a GAN-based Anomaly score (AS) for predicting cosmetic outcomes in breast cancer patients, we explored the long-term impact of radiotherapy (RT) on patients with permanent implants^5^. While our previous study demonstrated excellent predictive capability and highlighted the detrimental effects of RT on patients with tissue expanders compared to autologous reconstruction, it was limited to a single institution and focused on patients with tissue expanders at the time of analysis.

Therefore, to comprehensively evaluate the influence of RT on permanent implants, we validated the applicability of the previously developed GAN model. Additionally, we built a Normal Tissue Complication Probability (NTCP) model incorporating relevant clinical factors to predict the likelihood of major complications after RT.

## Materials and methods

We computed the AS using a previously established fast anomaly generative adversarial network (f-AnoGAN) model. The f-AnoGAN model consists of two primary components: a generator and a discriminator. The generator is trained to produce realistic medical images from random noise, while the discriminator is trained to distinguish between real and generated images. After training, the model identifies anomalies by comparing real images to generated ones, highlighting discrepancies. This approach leverages the unsupervised learning capabilities of GANs to efficiently identify and localize anomalies without the need for labeled data. As described in our previous research, the f-AnoGAN model was trained using normal breast images from 251 patients who underwent BCS^5^. The validation dataset consisted of 82 breast cancer patients who underwent immediate reconstruction surgery followed by radiotherapy between January 2016 and December 2020. This study received review and approval from the Institutional Review Board of Seoul National University Hospital (IRB No. 2304-009-1418).

For each patient, CT images were obtained at four different time points: RT simulation, the 1st follow-up, 1 year after completion of radiotherapy (Post-1Y), and 2 years after completion of radiotherapy (Post-2Y). We created 3D renderings for each patient using the Digital Imaging and Communications in Medicine (DICOM) images acquired from the CT scans. The isosurfaces of the 3D volumes were constructed and displayed in white with a white background and then resized to 500 × 500 pixels. All procedures were performed using MATLAB 2021a (The MathWorks Inc., United States). Next, at each time point, AS were calculated using the resized images with the previously published f-AnoGAN model (https://github.com/bigwiz83/SNUHRO_anoGAN_for_Cosmesis). The higher the Anomaly Score (AS), the more significant the anomaly detected, indicating a poorer cosmetic outcome.

**Figure 1:**
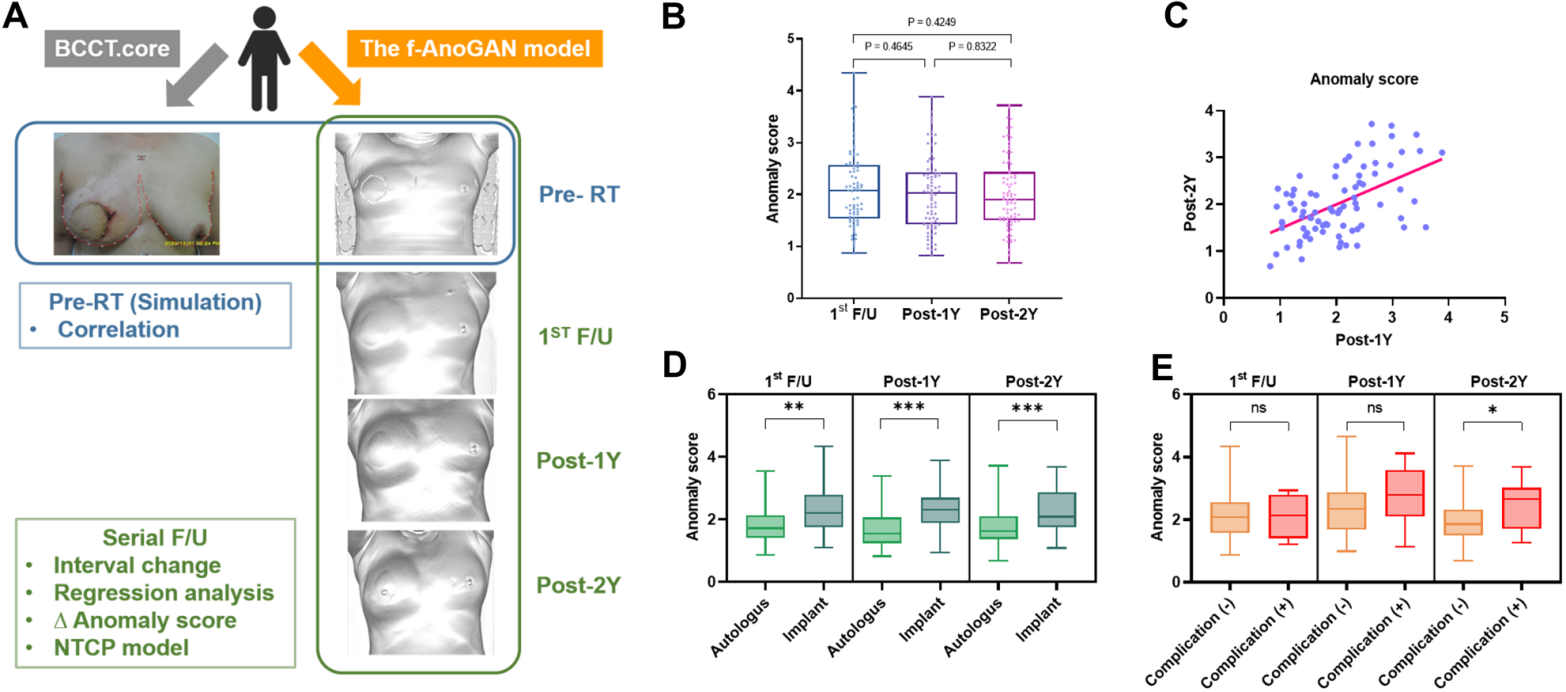
Overview of comparative analysis using developed f-AnoGAN model and BCCT.core software, and time-sequence comparison of AS. (**A**) Graphical representation of comparative analysis for BCCT.core and the f-AnoGAN model. (**B**) Pairwise comparison of anomaly score among 1st F/U, Post-1Y, and Post-2Y time points. (**C**) Relationship between AS of Post-1Y and Post-2Y. (**D**) Stratified by reconstruction type, anomaly scores according to three time points are compared. (**E**) In each time point, anomaly score is compared according to complication event. P-value was estimated by pairwise paired T-test. The 95% confidence intervals are drawn as error bars at each point. 3D 3-dimensional, f-AnoGAN Fast anomaly generative adversarial network, RT Radiation therapy, NTCP Normal Tissue Complication Probability, F/U follow-up, 1Y 1 year, 2Y 2 years.

Additionally, for each patient, pre-radiotherapy photographs of the entire bilateral breasts were captured from below the neck using a digital camera. Using BCCT.core software, we digitally placed markers at the sternal notch, 25 cm below the sternal notch, and along the entire contour of the breasts^6,7^. In patients without an ipsilateral nipple who underwent autologous reconstruction without nipple-areolar complex reconstruction, the nipple point was virtually marked symmetrically to that on the contralateral side. Objective geometric indices and cosmetic outcomes of the digital photographs were calculated using the BCCT.core software and categorized as excellent, good, fair, or poor. The detailed content of the analyses is specified in Fig. 1A.

Multivariate regression analysis was employed to examine the influence of clinical factors on both the interval increase in AS and the occurrence of complications. Additionally, a Normal Tissue Complication Probability (NTCP) model was constructed by fitting the probability of complications to a sigmoid function as follows:

$$NTCP= \frac{\text{exp}\left(logit(p)\right)}{1+\text{exp}(logit(p))}$$

Here, $p$ is denoted as the coefficient obtained from the regression analysis, the logit function is used to model the relationship between the clinical factors, and the probability of complications, and the sigmoid function is applied to transform the logit values into probabilities. Statistical analyses were conducted using Stata 17.0 (StataCorp LP, College Station, Texas). Correlation coefficient was achieved by simple regression analysis and graphs were generated using PRISM version 9.1.1.

### Ethics approval and consent to participate

This study received review and approval from the Institutional Review Board of Seoul National University Hospital (IRB No. 2304-009-1418). The requirement for written informed consent waive was approved by the Institutional Review Board of Seoul National University Hospital because individual information was analyzed anonymously.

## Result

### Patient characteristics

We reviewed the electronic medical records of 82 breast cancer patients who underwent immediate reconstruction surgery followed by radiotherapy. Patients who had undergone delayed reconstruction were excluded from this study. The median age was 46 years (range, 29–62 years). Body mass index (BMI) was ≤ 23 kg/m^2^ in 35 patients (42.7%), while BMI > 23 kg/m^2^ was in 47 patients (57.3%). Diabetes mellitus was reported in one patient (1.2%), and a history of smoking was observed in two patients (2.4%). The distribution of laterality was balanced, with an equal number of patients presenting with left-sided and right-sided breast cancer. Invasive ductal carcinoma was the predominant histology (n = 73, 89.0%). A minority of the cohort presented with an advanced pathologic T stage of pT3 or higher (n = 5, 6.1%) and an N stage of pN2 or higher (n = 18, 22.0%). Invasion to skin, nipple and muscle was present in 5 (6.1%), 2 (2.4%) and 1 (1.2%) patient, respectively. More than half of the cohort received skin-sparing mastectomy (n = 45, 54.9%). Axillary lymph node dissection was performed on 43 patients (52.4%), whereas sentinel lymph node biopsy alone was conducted in 39 patients (47.6%). Within the cohort, 38 patients (46.3%) received immediate implant insertion and 44 patients (53.7%) underwent autologous breast reconstruction. Additionally, tumor bed boost radiotherapy (n = 5, 6.1%), regional lymph node irradiation to the internal mammary nodes (n = 65, 79.3%), and supraclavicular lymph node irradiation (n = 68, 82.9%) were performed, based on risk factor stratification. For 29 patients with early-stage breast cancer (35.4%), the target volume was delineated according to the 2019 ESTRO-ACROP consensus guideline^8^. Detailed patient characteristics are presented in Table 1.

**Table 1:** Patient characteristics.

Variables	Total (N = 82)	
	No	(%)
Age (year)	46 (median) (range, 29‒62)	
Diabetes mellitus		
Yes	1	1.2%
No	81	98.8%
Smoking history		
Yes	2	2.4%
No	80	97.6%
BMI (kg/m^2^)		
> 23	35	42.7%
≤ 23	47	57.3%
Laterality		
Right	41	50.0%
Left	41	50.0%
Histology		
IDC	73	89.0%
ILC	7	8.5%
Others	2	2.4%
Pathologic T stage		
(y)pT0‒2	77	93.9%
(y)pT3‒4	5	6.1%
Pathologic N stage		
(y)pN0‒1	64	78.0%
(y)pN2‒3	18	22.0%
Invasion to skin		
Yes	5	6.1%
No	77	93.9%
Invasion to nipple		
Yes	2	2.4%
No	80	97.6%
Invasion to muscle		
Yes	1	1.2%
No	81	98.8%
Type of breast surgery		
Nipple-sparing mastectomy	33	40.2%
Skin-sparing mastectomy	45	54.9%
Total mastectomy	4	4.9%
Type of axillary surgery		
SLNB	40	48.8%
ALND	42	51.2%
Type of reconstruction		
Implant insertion	38	46.3%
Autologous reconstruction	44	53.7%
Axillary LN dissection		
Yes	43	52.4%
No	39	47.6%
Implant-saving target volume (2019 ESTRO-ACROP)		
Yes	29	35.4%
No	53	64.6%
RT to internal mammary LN		
Yes	65	79.3%
No	17	20.7%
RT to supraclavicular LN		
Yes	68	82.9%
No	14	17.1%
Tumor bed boost RT		
Yes	5	6.1%
No	77	93.9%
RT modality		
3D-conformal RT	1	1.2%
Fixed field IMRT	72	87.8%
VMAT	9	11.0%
BCCT.core grade		
Excellent	9	11.0%
Good	35	42.7%
Fair	37	45.1%
Poor	1	1.2%

ALND, axillary lymph node dissection; BMI, body mass index; IDC, invasive ductal carcinoma; ILC, invasive lobular carcinoma; IMRT, intensity-modulated radiotherapy; LN, lymph node; RT, radiotherapy; SLNB, sentinel lymph node biopsy; VMAT, volumetric modulated arc therapy.

### Relationship with BCCT.core grade and AS

Digital photographs taken prior to the initiation of radiotherapy were analyzed to objectively categorize cosmetic outcomes using BCCT.core software. The outcomes were classified as follows: excellent (n = 9), good (n = 35), fair (n = 37), and poor (n = 1). BCCT.core software was employed to calculate objective indices, namely Breast Retraction Assessment (BRA), Lower Breast Contour (LBC), Upward Nipple Retraction (UNR), Breast Compliance Evaluation (BCE), Breast Contour Difference (BCD), and Breast Area Difference (BAD). Among these indices, the grade of BCCT.core demonstrated a significant correlation with LBC, UNR, BCE, BCD, and BAD (P < 0.001, P < 0.001, P = 0.001, P = 0.003 and P = 0.043, respectively). Notably, BCD, which quantifies the disparity in the contour lengths of the left and right breasts, and BAD, which measures the difference in areas of the left and right breasts, both showed a significant association with the AS at simulation CT (P = 0.009 and P = 0.004, respectively) (Supplementary Table 1).

### Temporal characteristics of AS

**Figure 2:**
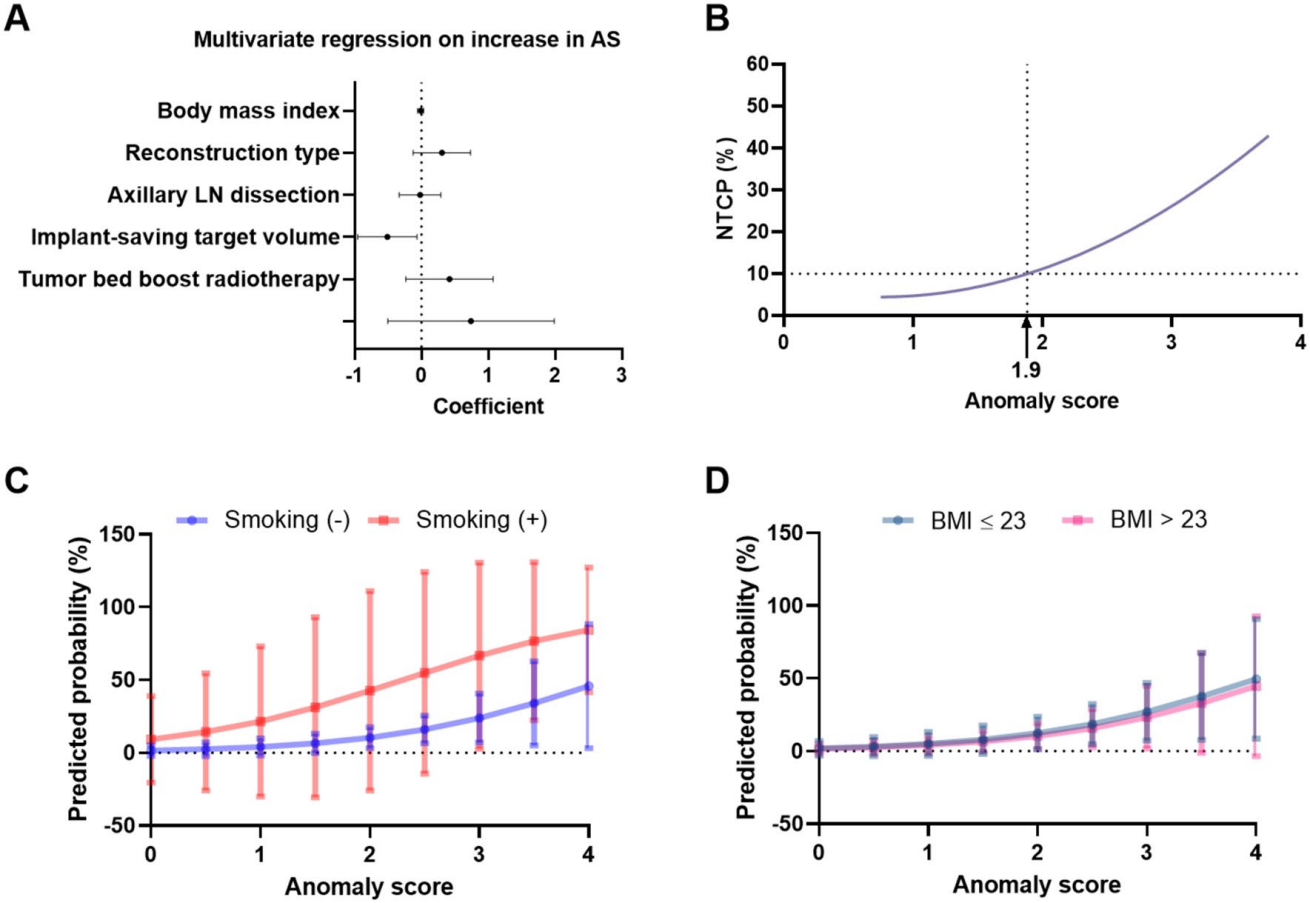
Multivariate analysis on increase in AS and predicted complication probability according to variables. (**A**) Multivariate regression analysis on increase in AS. (**B**) Probability of complication upon NTCP model. (**C**) Comparison of predicted probability of complication according to smoking history and **D** BMI status. The 95% confidence intervals are drawn as error bars at each point. LN lymph node, NTCP normal tissue complication probability, BMI body mass index.

During the serial follow-up, AS from the 1^st^ follow-up, Post-1Y, and Post-2Y exhibited no significant variations across these time intervals (Fig. 1B). However, a notable positive correlation was observed between the AS values of Post-1Y and Post-2Y, with a correlation coefficient of 0.515 (P < 0.001, Fig. 1C). Subgroup analysis revealed that AS was significantly elevated in patients who had received prosthetic implant insertion as compared to those who underwent autologous reconstruction at the 1st follow-up (P = 0.001), Post-1Y (P < 0.001) and Post-2Y (P < 0.001) (Fig. 1D), indicating a poorer cosmetic outcome for those with implants than for those with autologous tissue reconstruction. Additionally, patients experiencing major complications demonstrated a significantly higher AS at Post-2Y (P = 0.020), but not in 1st F/U (P = 0.760) and Post-1Y (P = 0.128) (Fig. 1E). The median time interval from RT to major complication was 15.4 months. In multivariate regression analysis, patients treated with an implant-saving target volume showed a lesser increase in AS between Post-1Y and Post-2Y (Coefficient = 0.507, 95% CI − 0.950 to − 0.06, P = 0.025) (Fig. 2A). However, BMI, type of reconstruction, axillary lymph node dissection, and tumor bed boost RT did not affect the temporal change in AS between 1 and 2 years after RT.

### Construction of NTCP model

A multivariate logistic regression analysis was performed incorporating AS at Post-2Y as well as several well-known clinical factors associated with major complications including smoking history and BMI (Supplementary Table 2). We identified a significantly positive association with AS at Post-2Y (Coefficient 0.99, 95% CI 0.039‒1.974, P = 0.041) (Fig. 2B). However, factors such as smoking history (P = 0.206) and BMI > 23 (kg/m^2^) did not correlate with an increased likelihood of complications (P = 0.206 and P = 0.776, respectively). Given the significant correlation between AS and complications, the NTCP model was developed by fitting a sigmoid function with coefficient of 0.99 multiplied by AS at Post-2Y. The model is presented graphically in Fig. 2B, and NTCP model demonstrated that 10% of major complication was reached when AS at Post-2Y is greater than or equal to 1.9. As expected in the regression model, the predicted probability of complications showed no significant difference between patients stratified by smoking history (Fig. 2C) or BMI (Fig. 2D).

## Discussion

Our study evaluated the performance of a previously published GAN-based cosmetic evaluation tool in an external dataset of breast cancer patients who underwent RT. We found that the tool's output was positively correlated with the BCD and BAD, which are considered critical metrics of breast cosmesis. In addition, we found that the temporal change in AS was not significant in this dataset. However, the change in AS between 1 and 2 years after RT was less pronounced when implant-saving RT was adopted. Finally, we developed the NTCP model to predict the probability of major complications related to AS.

BCCT.core utilizes seven asymmetry features (BRA, LBC, UNR, BCE, BCD, BAD, BOD), along with their dimensionless counterparts (pBRA, pLBC, pUNR, pBCE, pBCD, pBAD, pBOD) and color difference features to classify cosmetic outcomes into four grades: excellent, good, fair, and poor. In our cohort, only 10 patients were categorized as either excellent or poor, indicating that the majority (n = 72) were classified as good or fair. Our analysis revealed that most BCCT.core indices did not demonstrate a direct correlation with AS. However, BCCT.core's intended use applies solely to patients undergoing breast-conserving surgery, distinct from our study population who all received mastectomy and reconstruction. Also, this may be attributed to the limited number of patients with extreme outcomes (excellent or poor) in our cohort, suggesting the need for optimizing the cut-off points used for grade stratification. Notably, BCD and BAD showed strongly positive correlation to the AS. Since these two indices refer to the difference in the cosmesis between right and left breasts, we may speculate that those are substantially important factors for cosmetic outcome and quality of life in patients receiving mastectomy and reconstruction^9^. Thus, it is plausible that AS showed strong correlation with both of BCD and BAD.

Moreover, a significant positive correlation was observed between Post-1Y and Post-2Y AS values, indicating that patients with poor cosmetic outcomes after mastectomy were likely to maintain this level of cosmesis following postmastectomy RT (PMRT). The detrimental impact on cosmesis, including complications such as capsular contracture and the necessity for revision surgeries after PMRT, has been well-known, as highlighted in several studies^5,10^. However, our findings suggest that patients with less optimal cosmetic outcomes after mastectomy may retain those outcomes even after PMRT. This highlights the crucial role of achieving excellent cosmesis during initial reconstruction surgery for long-term aesthetic success.

PMRT is known to pose risks for adverse cosmetic outcomes like capsular contracture or wound dehiscence^11^. A retrospective study^12^ reported poorer cosmetic outcomes in patients with higher BMI as assessed by BCCT.core software factor^13,14^. Recent literature identifies smoking and BMI as significant prognosticators for cosmetic outcomes, with a well-established link between smoking and postoperative complications^15^. When adjusting BMI and smoking status, we found that the Post-2Y AS was significantly higher in patients who experienced major complications. This may address the importance of cosmetic outcome as a predictive value for major complications. According to our NTCP model, complications were exceeded 10% of patients when AS was higher or equal to 1.9. Surpassing a threshold score of 1.9 is indicative of a sharp increase in the probability of complications; this model therefore provides a basis for early detection of adverse signs and potential intervention.

The influence of reconstruction type and complications on cosmetic outcomes were further explored in the subgroup analysis. Our previous analysis^1^ already revealed that the superiority of autologous reconstruction over tissue expander insertion. In current analysis, superior cosmetic outcomes were observed consistently in each time point in patients having autologous reconstruction compared to permanent implant. A review^16^ addressed that autologous tissue is more resistant to irradiation over implant-based material. Jagsi et al.^17^ reported lower risk of complications in patients who had undergone autologous reconstruction than implant-based reconstruction (Odds ratio 0.47, 95% CI 0.27—0.82, P = 0.007). However, there are several advantages for implant-based reconstruction such as shorter operations, hospital stays, and recovery time, or no scars to the donor site^18^, which is enabling persistent demand on implant-based reconstruction. Therefore, the importance of delineating implant-saving target volume has been arising^8^. In our study, adoption implant-saving target volume was significantly correlated with a lesser change of AS, meaning the minimal difference in cosmesis along with the time and supporting the efficacy of implant-saving contouring in selected patients.

This study has several limitations. First, due to the presence of attached scar markers at the time of the simulation for RT, accentuating the scars themselves. The 3D rendered images were highly sensitive to an exaggerated appearance of the scar. Thus, this may lead to the higher AS in the pre-RT, compared with the 1st F/U, Post-1Y and Post-2Y. Although the relatively small sample size was analyzed, it should be noted that this analysis was external validation of previously published model. Still, long-term follow-up data would solidify the role of anomaly GAN-based model in terms of evaluating cosmesis.

In conclusion, we validated the feasibility of the previously developed GAN-based model in terms of evaluation cosmesis in patients having mastectomy and reconstruction. We quantified the cosmesis as a AS and proposed the potential for predicting complications using the NTCP model. This tool was expected to objectively evaluate cosmetic outcomes and quality of life in breast cancer patients.

### Supplementary Information

Supplementary Tables.

## Data Availability

The datasets analyzed during the current study will be made available upon request after the publication of this manuscript. Please contact JBS (bigwiz83@gmail.com) for the data.
