# Supplementary material for: Evaluation of cosmetic outcomes in breast reconstruction patients undergoing radiotherapy using an anomaly generative adversarial network model
Source: Sci Rep. 2024 Jul 10;14:15940. doi: 10.1038/s41598-024-66959-1 (PMC11237143; doi:10.1038/s41598-024-66959-1)
Supplement: Supplementary file 1 — Supplementary Tables. [file 41598_2024_66959_MOESM1_ESM.docx]

| **Supplementary Table 1. Pearson correlation with objective indices from BCCT.core software and AS** | | | | | | | | |
| --- | --- | --- | --- | --- | --- | --- | --- | --- |
|  | AS | BCCT | BRA | LBC | UNR | BCE | BCD | BAD |
| **AS** | 1.000  ( - ) |  |  |  |  |  |  |  |
| **BCCT** | 0.017  (0.840) | 1.000  ( - ) |  |  |  |  |  |  |
| **BRA** | -0.093  (0.451) | 0.143  (0.244) | 1.000  ( - ) |  |  |  |  |  |
| **LBC** | 0.146  (0.235) | 0.590*  (< 0.001) | 0.090  (0.464) | 1.000  ( - ) |  |  |  |  |
| **UNR** | 0.080  (0.519) | 0.649*  (< 0.001) | 0.035  (0.776) | 0.608* (< 0.001) | 1.000  ( - ) |  |  |  |
| **BCE** | 0.019  (0.880) | 0.407*  (0.001) | 0.068  (0.580) | -0.105  (0.394) | 0.542*  (< 0.001) | 1.000  ( - ) |  |  |
| **BCD** | 0.316*  (0.009) | 0.361*  (0.003) | 0.136  (0.268) | 0.693*  (< 0.001) | 0.547*  (< 0.001) | -0.039  (0.754) | 1.000  ( - ) |  |
| **BAD** | 0.343*  (0.004) | 0.246*  (0.043) | 0.200  (0.103) | 0.483*  (< 0.001) | 0.429*  (0.000) | 0.030  (0.810) | 0.824*  (<0.001) | 1.000  ( - ) |
| Abbreviations: BRA, Breast retraction assessment; LBC, Lower breast contour; UNR, Upward nipple retraction; BCE, Breast compliance evaluation; BCD, Breast contour difference; BAD, Breast area difference.  P-values are shown in parentheses. | | | | | | | | |
|  |  |  |  |  |  |  |  |  |
|  |  |  |  |  |  |  |  |  |

| **Supplementary Table 2. Multivariate regression on major complication** | | |
| --- | --- | --- |
| **Variables** | **Coefficient (95% CI)** | **P value** |
| AS at Post-2Y | 0.99 (0.04–1.95) | 0.041 |
| Smoking history | 1.86 (-1.02–4.73) | 0.206 |
| BMI ≤23 | -0.20 (-1.58–1.18) | 0.776 |
| cons | -4.03 (-6.64– -1.41) | 0.003 |
